# Study of the Influence of Bamboo Suspension Water-Removal Processes on the Properties of Bamboo-Based Molding Materials

**DOI:** 10.3390/polym16233337

**Published:** 2024-11-28

**Authors:** Xiaowei Zhuang, Weichen Li, Xin Pan, Hui Qiao, Baoyong Liu, Weiming Yang, Yongshun Feng

**Affiliations:** 1 Zhejiang Academy of Forestry, Liuhe Road 399, Hangzhou 310023, China; zhuangxiaowei@zjforestry.ac.cn (X.Z.); panxin@zjforestry.ac.cn (X.P.); yangweiming@zjforestry.ac.cn (W.Y.); 2 College of Environmental Science and Engineering, Liaoning Technical University, Zhonghua Road 47, Fuxin 125105, China; f050914411@163.com (W.L.); liubaoyong00@163.com (B.L.)

**Keywords:** bamboo fiber, hydrated thermochemical grinding, porous, molding materials

## Abstract

Bamboo is a fast-growing lignocellulosic plant in nature. It is an abundant and renewable resource with wide applications. The processing of bamboo results in a large amount of residue. In this paper, we developed a method to utilize bamboo residue to prepare a novel lightweight porous molding material. A hydrated thermochemical grinding process was proposed to disintegrate bamboo fibers and activate bamboo’s own binding components. The influence of the water removal by pressure from bamboo suspension and subsequent different drying methods on the product’s properties was evaluated. The two-step drying ensured a low production cost and high product quality. The bamboo molding material was characterized based on thermal stability, morphology, functional groups, particle size distribution, crystallinity, and mechanical strength. A lightweight porous material was obtained with a density of 0.23–0.35 g/cm^3^ by freeze-drying. A high mechanical strength was obtained with a tensile strength of 0.62 MPa and a compressive strength of 10.31 MPa by oven drying. The auto-adhesive mechanisms, including fiber anchorage, polymerization, water plasticization, and heat plasticization, were discussed. The bamboo molding material is a reconstruction of bamboo cell wall components and is easy to recycle. It has potential applications in construction and buildings, packaging, and indoor furnishings.

## 1. Introduction

Porous materials are defined as a kind of dispersion in which a large proportion of gas is dispersed in a liquid, solid, or gel. They have useful properties such as low density and a large surface area, and have proved to be an essential part of our daily life [1]. A typical example of porous material is plastic foam made from petrochemical feedstocks including polyurethane, expanded polystyrene, polyvinyl chloride, etc. [2]. The large-scale application of plastics and inadequate recycling could cause the contamination of the environment in the earth system [3]. The natural lignocellulosic foams are new-generation lightweight porous materials showing adequate mechanical strength, thermal conductivity, and sound adsorption. They consist of renewable resources that are climate-friendly and recyclable. They can be used in products such as thermal insulation, heat exchangers, acoustic absorption, oil adsorbents, etc.

In earlier research, several attempts were made to develop lightweight insulation materials by using lignocellulosic feedstock since the 1940s. A Swedish paper and pulp factory used residues of the sulfite pulp production and alkaline liquor to make highly porous fiberboards with extremely low densities of 0.06–0.08 g/cm^3^ [4]. The production was not taken forward due to the competition from polyurethane at that time. The development of foamed wooden materials once again attracted the attention of many researchers in the new millennium. Wooden flour, wheat or grain, and yeast were mixed to make porous materials by fermentation [5,6]. The resulting porous bio-based boards had a bulk density of 0.34 g/cm^3^ with excellent mechanical performance.

In recent studies, wood foam and wood sponge are the newest and frontmost topics in the field of wood science and technology. Aiming at oil absorbents, the spring-like wood sponge was prepared by removing the lignin with acid solution and hemicelluloses with alkali solution from the cell wall followed by freeze drying [7]. As a lightweight thermal insulation material, a biomimetic swallow nest structure was assembled by using bamboo scraps as the supporting framework and methylcellulose as the templates. The lightweight composite had a density of 0.42 g/cm^3^ with a high compressive strength of 6.5 MPa [8]. The waste wood chips were used to produce insulation foam through the alkali delignification process by foaming with carboxymethylcellulose sodium salt. The wood-based foam had a density of 0.06 g/cm^3^ and a thermal conductivity of 0.038 W/(mK) [9]. The unrefined pine beetle-killed wood was used to produce lightweight solid foams with a density of 0.12 g/cm^3^ and thermal conductivity of 0.042 W/(mK) [10]. The wastepaper-based foam was prepared by the lignocellulosic fibers coupled with PVA and gelatin. The resulting density was 0.06–0.08 g/cm^3^ and the thermal conductivity was 0.044 W/(mK) [11]. Significant progress in bio-based foams has been made owing to the growing demand for green and sustainable products [12].

The conventional methods to make lignocellulosic lightweight porous materials usually require the delignification process, binders, foaming agents, and high molecular weight polymers. The complicated processes result in high-performance porous materials but lead to complexity, high cost, and recycling issues. Furthermore, the entire utilization of biomass still needs to be augmented since lignin is an unwanted constituent in the traditional methods [13]. Lignin, as nature’s dominant aromatic polymer, is found in most plants in the range of 15–40% and provides structural integrity. It is an encrusted component and acts as the glue that binds cellulose and hemicelluloses together [14]. Multiple strategies need to be developed to make full use of these natural aromatic polymers [15]. A recent study confirmed that the uncondensed or slightly condensed lignin had superior adhesive properties when applied to the plywood [16]. The milled wood lignin was isolated from eucalyptus in a planetary ball mill at 600 r.p.m. for 10 h and extracted with 96% dioxane solution. The prepared plywood with superior performance enabled the use of lignin adhesives as promising alternatives to traditional formaldehyde-based adhesives. The concept of activating and utilizing wood’s own bonding strength was also proposed by researchers at Fraunhofer Institute for Wood Research Wilhelm Klauditz Institut (WKI) in Germany for the preparation of porous, pressure-resistant material from renewable raw materials [4,17]. The production of so-called “wood foam” generally involves four steps: (1) the production of wood chips; (2) the refinement of wood chips to wood fibers; (3) the preparation of wood-water suspension with highly fibrillated fibers; and (4) foaming and drying [18]. The wood foam has a density of 0.04–0.28 g/cm^3^ with a compressive strength from 20 to 600 kPa. The WKI process requires a costly thermochemical defibrillator and refiner. From this perspective, a low-cost hydrated thermochemical grinding process (HTG process) was successfully developed to obtain auto-adhesive suspensions [19]. A lightweight bio-degradable lignocellulosic porous molding material was obtained with a density in the range of 0.28–0.67 g/cm^3^.

Bamboo is a fast-growing plant that is the largest member of the grass family but has wood fibers. It is widely used as a raw material for charcoal, tableware, furniture, flooring, etc. The processing of bamboo leads to a large amount of residue, such as bamboo chips and bamboo powder. In this study, bamboo powder was used to develop a novel bamboo-based porous molding material. An improved HTG process was proposed to activate the bamboo’s own binding components. A two-step drying method was developed to balance the production cost and product quality. This study provided a green alternative way to make full use of bamboo residue.

## 2. Materials and Methods

### 2.1. Materials

Raw materials of bamboo powder were purchased from Zhumeng Bamboo Technology Company (Xuancheng, China). As a kind of residue from bamboo timber processing, the main chemical constituents of bamboo powder in general are cellulose (60–70%), hemicellulose (20–30%), lignin (20–30%), and pentosans (20–25%) [20]. Bamboo powders were sieved with 20 mesh (0.85 mm) to remove impurities and obtain a uniform size. They were dried at 60 °C for 4 h and stored in plastic bag to avoid mildew.

### 2.2. Preparation of Bamboo-Based Porous Molding Materials

**Figure 1:**
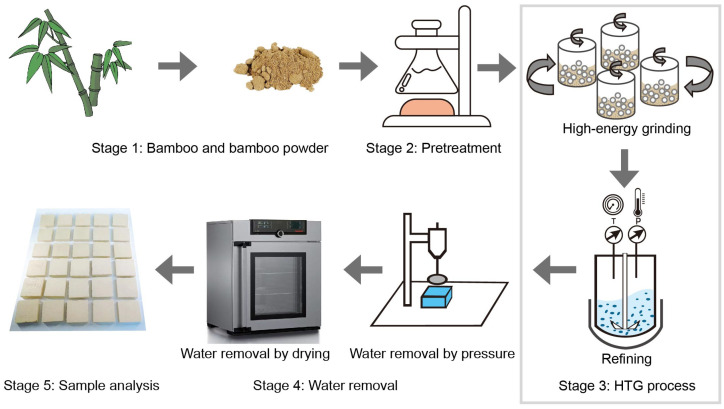
Schematic illustration of the preparation of bamboo-based molding materials.

The bamboo-based porous molding materials were prepared through a hydrated thermochemical grinding process shown in Figure 1. The bamboo powder was boiled in a 0.05 wt.% NaOH solution for 1 h and washed with deionized water until reaching a neutral pH. The washed bamboo powder was dried at 60 °C for 4 h.

A total of 30 g pretreated bamboo powder was mixed with 90 g deionized water and placed into a 500 mL stainless-steel grinding pot (Model 3SP2, Nanda Instrument, Nanjing, China). The grinding time was 2 h. After hydrated grinding, the resulting bamboo-water suspension was introduced into a high-pressure reactor (Model 4547, Parr Instrument, Moline, IL, USA) for further defibration. The reaction time was set as 30 min with a rotating speed of 600 r.p.m., pressure of 0.6 MPa, and temperature of 120 °C. The refined bamboo-water suspension was collected and foamed with an intensive mixer. The foamed suspension was then poured into a stainless-steel parallelepiped mold with the size of 7 cm × 7 cm × 5 cm. A wooden lid which was a little bit smaller than the mold was put on the suspension. A pressure of 0, 20, 80, 140, and 200 kPa was added to the lid, respectively, for 20 min to remove the water from the bamboo roughcast. The mold was removed before drying.

Two drying methods, namely, oven drying and freeze drying, were employed to remove the remaining water. For the oven drying, the temperature was set at 80 °C and kept for 15 h. The weight of all samples was recorded every 1 h to track the dynamic weight loss. For the freeze drying, samples were put in the freeze dryer (SCIENTZ-12N/A, Ningbo, China) for 5 days and the weight was recorded after every 24 h.

### 2.3. Distribution of Fiber Length and Particle Size in Bamboo Suspension

The distribution of particle size was measured by the laser particle size analyzer (Malvern Mastersizer 2000E with the software Mastersizer v3.81, Malvern, UK). Before measuring, the bamboo suspension was filtered by a 100 mesh (0.15 mm) screen. HydroEV was used as a standard operation program. The shading degree was set at around 6%. The refractive index of particles was 1.530 and the absorption factor was 0.1. Water was used as the dispersing agent with a refractive index of 1.330. All the tests were conducted 3 times and the average value was used for the analysis.

### 2.4. Mechanical Strength of Bamboo-Based Porous Molding Samples

The mechanical strength of the samples was conducted according to test methods for the physical and mechanical properties of small clear wood specimens. Before making the test specimen, the top surface layer of all the samples was removed by a sander to achieve a uniform performance. The shape of samples was made into cuboid with the removal of the surface. The samples were dried in the oven and the oven-dry density was obtained by measuring the volume and the weight (GB/T 1927.5-2021) [21]. The tensile strength, compressive strength, bending strength, and Young’s modulus were conducted by a universal mechanical testing machine (WSM-30KN, Changchun Intelligent Instrument Equipment Co., Ltd., Changchun, China) according to GB/T 1927.14-2021 [22]. The impact energy was measured by an impact testing machine (JJ-15, Changchun Intelligent Instrument Equipment Co., Ltd., Changchun, China) according to GB/T 1927.17-2021 [23]. The shore hardness was conducted by a shore hardness tester (LandTek, Guangzhou, China) according to GB/T 1927.19-2021 [24]. The measurements were performed in triplicate and the average value was used for the analysis.

### 2.5. Thermal Stability of Bamboo After HTG Process

The thermal behavior of the samples was determined using a thermogravimetric analyzer (Q500, TA Instruments, New Castle, DE, USA). For each test, samples of around 10 mg were placed in the crucible and heated from the ambient temperature to 800 °C. The heating rate was set at 15 °C/min. The experiment was conducted under a high-purity nitrogen atmosphere with a flow rate of 40 mL/min.

### 2.6. FTIR Analysis of Bamboo After HTG Process

The infrared spectra of the bamboo raw material and the suspension after HTG process were recorded via FTIR (Nicolet iS 5, Thermo Fisher Scientific, Waltham, MA, USA) using the KBr pressed-pellet technique. The samples were dried at 103 °C to remove the free water and the operations were done under an infrared baking lamp. The sample powder was mixed with KBr with a weight ratio of 1:100 and pressed into transparent pellets. The spectra were recorded from 32 scans over the wavenumber range between 400 and 4000 cm^−1^. The resolution of the spectrometer was 2 cm^−1^. The baseline was calibrated before each test.

### 2.7. XRD Analysis of Bamboo After HTG Process

The crystalline structures of bamboo raw material and bamboo suspension was determined via X-ray diffraction (Rigku SmartLab, Akishima, Japan) with Cu Kα radiation (wavelength λ = 0.154 nm). The scanning range was set at 2θ = 5–40°, the scanning step at 0.02°, scanning speed at 4°/min, the tube voltage at 40 kV, and the tube current at 30 mA. The crystallization index was calculated using Segal’s empirical formula [25]:

C_rI_ = 100% × (I_002_ − I_am_)/I_002_

where C_rI_ is the crystallinity index (%), I_002_ represents the diffraction intensity of the 002 crystalline plane (2θ = 22°), and I_am_ represents the diffraction intensity of the amorphous region (2θ = 18.0°).

## 3. Results and Discussion

### 3.1. Pyrolysis Characteristics of Bamboo Treated by HTG Process

**Figure 2:**
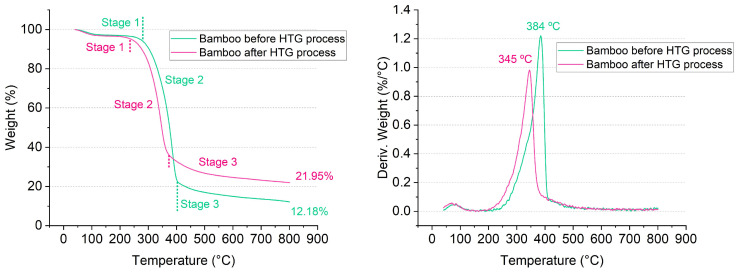
Pyrolysis characteristics of bamboo before and after HTG process.

As a kind of lignocellulosic biomass, bamboo showed a typical pyrolysis process with three weight-loss stages despite the HTG process [26]. The pyrolysis characteristics of bamboo before and after the HTG process at a heating rate of 15 °C/min are shown in Figure 2. However, the temperature range and weight loss of each stage had been changed owing to the HTG process, shown in Table 1.

The first stage up to a temperature of 280 °C was the release of water molecules bonded through physical links to the lignocellulosic substrate. The highest weight loss of raw bamboo sample occurred in the second stage from 280 to 400 °C with the degradation of cellulose, hemicellulose, and, also, the partial lignin. Liang et al. divided bamboo into the outer layer, middle layer, and inner layer, which showed a second pyrolysis stage corresponding to the temperature of 200–400 °C with around 70 wt.% of weight loss [27]. The second stage of bamboo after the HTG process corresponded to the temperature of 235–370 °C, which was 45 °C lower than that of bamboo raw material. The maximum weight-loss rate of bamboo after the HTG process was 345 °C, which was also 39 °C lower than that of bamboo raw material. The shift to the left side of both the TG and DTG curves indicated a susceptible thermal degradation of bamboo after the HTG process. In this study, the HTG process played a key role as it activated bamboo’s own binding components while multiscale bamboo fibers were also released in this process. The outside wall temperature of the stainless-steel grinding pot was ca. 65 °C. The temperature inside the grinding pot was much higher than that of the outside wall with a certain pressure. Due to the high energy grinding, the instant temperature between grinding balls could even reach several hundred degrees Celsius. The glass transition temperature of cellulose crystals is greater than 243–307 °C following the decomposition of cellulose crystals [28]. The grinding pot worked as a reactor, in this case, with a high revolution speed. The bamboo powder underwent thermal degradation during the HTG process.

The third stage was from 400 to 800 °C for bamboo and 370 to 800 °C for bamboo after the HTG process. The weight loss in the third stage was 10.47 wt.% for bamboo and 14.80 wt.% for bamboo after the HTG process. A large number of oligomers and low molecular weight compounds were formed in the bulk of the lignocellulosic material during the HTG process. Therefore, the bamboo after the HTG process had a higher weight loss due to the degradation caused by HTG processing. The main reaction in this stage was the degradation of lignin, tar, and char [29]. The final residue was 12.18 wt% for bamboo raw material and 21.95 wt.% for bamboo after the HTG process. Under the action of high-energy mechanical forces, a certain content of iron was transferred from the stainless-steel grinding medium into the lignocellulosic suspension, leading to a high amount of the final residue [19].

### 3.2. FTIR and XRD of Bamboo Treated by HTG Process

**Figure 3:**
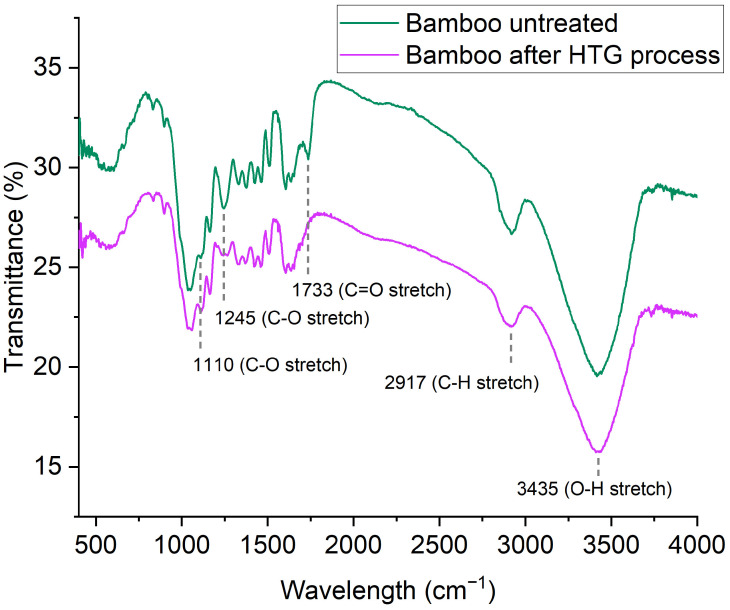
FTIR spectra of bamboo samples.

The changes in the functional groups in bamboo after the HTG process were shown by FTIR spectral regions between 400 and 4000 cm^−1^, as shown in Figure 3. The peak at 3435 cm^−1^ represents the stretching vibration of the O-H group. A significant change in FTIR spectra was found at 1733 cm^−1^, which represents the stretching vibration of the C=O group. It is often attributable to free carboxyls and the stretching of acetyl carboxylic acids in hemicellulose [30]. Peaks at 1245 and 1110 cm^−1^ represent the stretching vibration of the C-O group, which is attributed to esters and guaiacyl ring breathing in lignin [30]. The decrease in these peaks indicated a change in the C=O conjugated structure and C-O structure of bamboo material.

During the HTG process, it was speculated that the degradation of cellulose and hemicellulose formed various oligo- and/or monosaccharides. The saccharides were further converted into aldehydes, pyrans, furans, etc. Meanwhile, part of the lignin was degraded into a soluble fraction composed of guaiacyl and syringyl monomers and oligomers. The lignin soluble fraction was stabilized by aldehydes originating from saccharides [31]. Complex chemical reactions took place during the drying process above 80 °C, involving the softening of uncondensed lignin by water and the polymerization of low molecular weight phenolic compounds [16]. The chemical reactions in the HTG process and the following drying process contributed largely to the mechanical performance of the bamboo-based porous molding materials.

**Figure 4:**
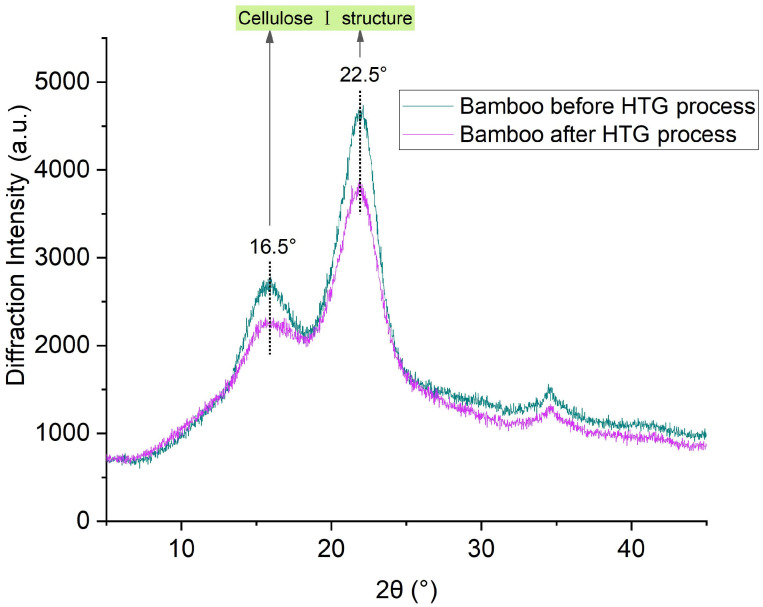
X-ray diffraction of bamboo samples.

Figure 4 shows the X-ray diffraction and crystallinity indexes of the bamboo samples. The peaks at 16.5 and 22.5° indicate a typical cellulose I structure [32]. It is the native form of cellulose in plant cell walls and the most abundant form in nature. Cellulose I has a two-dimensional layered structure with extensive inter- and intra-molecular hydrogen bondings. The scission of hydrogen bonds in Cellulose I occurs in the temperature range of 40–220 °C, forming furfural and 1,4:3,6-dianhydro-α-D-glucopyranose [28]. According to Figure 4, the cellulose I structure of bamboo was observed after the HTG process. However, the decrease in the crystallization peaks showed a decrease in the content of the cellulose I. The amorphous cellulose was possibly converted to the crystalline fraction during the HTG process [33]. The evaluation of the degree of crystallinity was based on the area of the peak (002) [34]. The crystallinity index of bamboo raw material was 30.6% while that of bamboo after the HTG process was 23.6%. The high crystallinity showed that a large part of the crystalline structure of cellulose in bamboo fibers remained after the HTG process. The preserved crystalline part of cellulose was supposed to provide physical strength to the molding samples.

### 3.3. Morphology of Bamboo Fiber Produced During HTG Process

**Figure 5:**
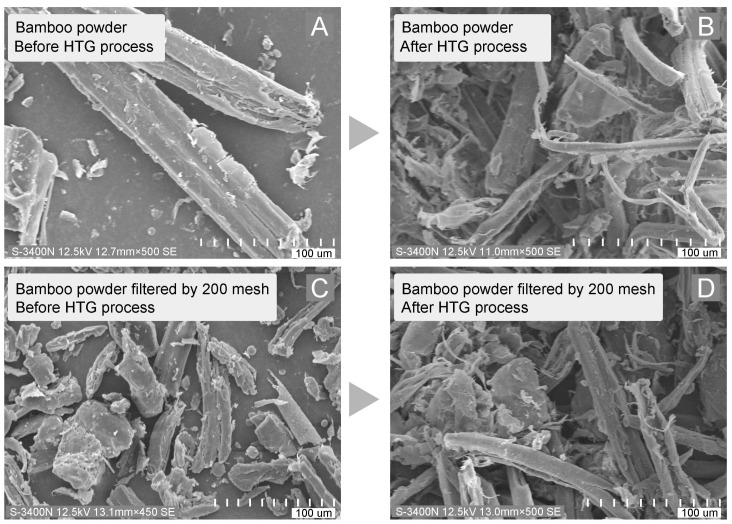
SEM images of bamboo powder before and after HTG process.

The manufacturing of bamboo-based porous molding materials included several steps, among which the HTG process played a crucial role. Besides the degradation of original lignin and the formation of uncondensed lignin, bamboo fibers underwent disintegration during the HTG process. As shown in Figure 5A,C, the raw bamboo powder had a smooth surface and clear patterns. After HTG process, the bamboo fibers became rough and branched due to the high-energy grinding shown in Figure 5B,D. The high-energy HTG process caused the breakage of macromolecular chains and intra- and intermolecular bonds, leading to the formation of oligomers with different lengths and various branches with reactive end groups. Meanwhile, the low molecular weight compounds were formed primarily from the thermo- and mechano-chemical degradation process and secondarily from the recombination and reactions of various reactive species produced during HTG process. The resulting rough and branched fibers had an anchorage effect. They tangled with each other and hung on the fibers to form a high viscosity suspension. This is like a bird’s nest structure with light weight and porous property. Compared to similar research, our study focused totally on the bamboo’s own binding components rather than the use of vinyl acetate/ethylene [8].

During the HTG process, the intensive crushing and rubbing under a certain temperature and pressure caused fibrillation of the bamboo cell wall. The surface area largely increased, providing more sites for the activated natural adhesive components. Along with the released constituents, the high viscosity suspension had good gas holding capacity, which was able to form a porous structure. The physical links and chemical bonding contributed to the cohesion of the solid form and enhanced the mechanical performance of the final products.

**Figure 6:**
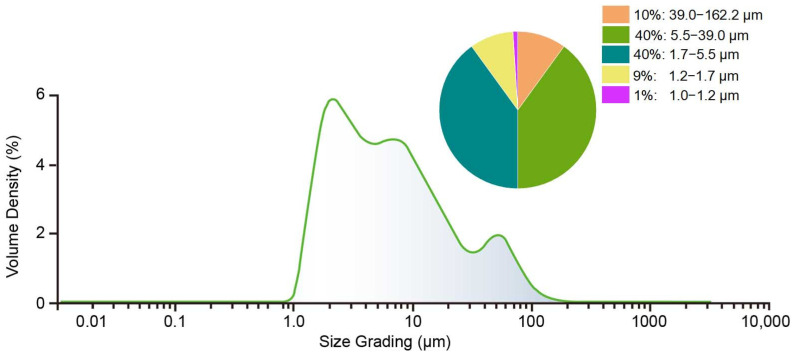
Distribution of particle size in the bamboo suspension after HTG process.

The bamboo raw material was disintegrated into fibers, after the HTG process, with various sizes. The distribution of the particle size is shown in Figure 6. The suspension was filtered beforehand through a 100-mesh screen to remove fibers longer than 150 μm. In the range of 1–1000 μm, which was detectable by laser particle size analyzer, fibers with a size between 1.7 and 5.5 μm accounted for 40%, and those between 5.5 and 39.0 μm also accounted for 40%. This indicated that below the size of 162 μm, the majority of the bamboo fiber length was between 1.7 and 39.0 μm. The fiber length is closely related to the structure and mechanical strength of the final products. Long fibers are helpful in improving the internal bonding strength. Short fibers provide more contact points but lead to a high density of the products. The formation of a reasonable matrix with nanoscale, microscale, and macroscale fibers is attractive for future study.

### 3.4. Water Removal and Molding of Bamboo-Based Porous Material

Drying was generally the last step to prepare such porous molding materials. It played a crucial role in the production process as more than 80 wt.% of water needed to be removed. The drying method and speed significantly influenced the shape and mechanical performance of lignocellulosic-based porous molding materials. In order to evaluate the influence of the residual water content of bamboo suspension after pressing, the roughcast made from bamboo suspension with different water content was prepared and dried by oven drying and freeze drying, respectively, in this work.

**Figure 7:**
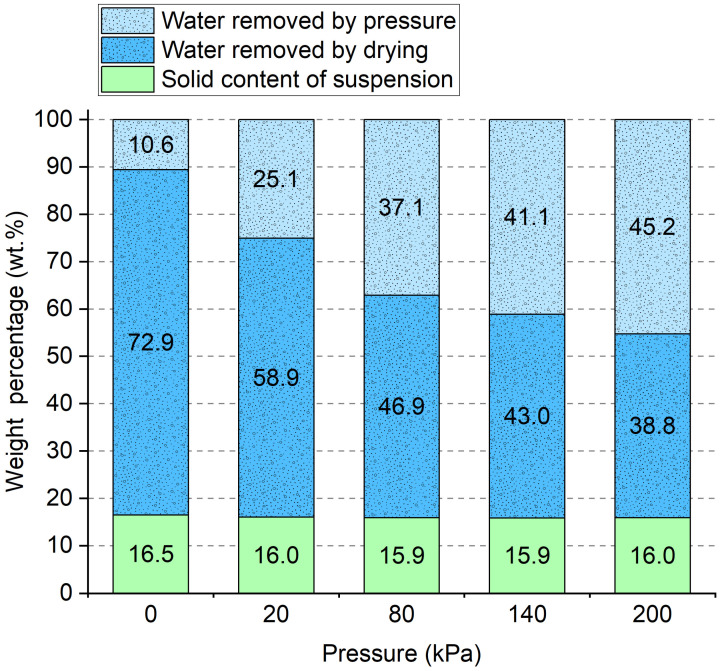
Water removal with different pressures.

After the HTG process, a high-viscosity bamboo-based suspension was formed but with high water content. The suspension had a water content of 83.5 to 84.1 wt.%, which needed to be removed afterwards. During the drying process, water was responsible for the energy consumption and plasticizing effect. To determine the influence of the water in the suspension on the properties of the final solid products, we compressed the roughcast in the mold with a pressure of 0, 20, 80, 140, and 200 kPa to reduce the moisture content as the first step in removing the water. The effect of water removal by pressure and subsequent drying from the porous bamboo mold is shown in Figure 7. Even without pressure, the roughcast in the mold had a weight loss of 11 wt.% after 12 h affected by gravity forces. As the pressure increased from 20 to 200 kPa, the weight percentage of removed water increased from 25.1 to 45.2 wt.%. The most significant reduction of water occurred from 20 kPa. Above 80 kPa, the percentage of water removal by pressure reached a maximum of 45.2% at 200 kPa but a much lower increasing rate.

**Figure 8:**
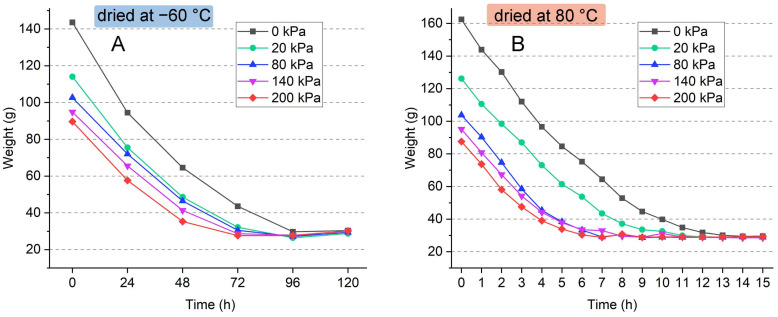
Weight loss of porous bamboo mold after freeze drying at −60 °C (**A**) and drying at 80 °C (**B**) as a function of time and pressure.

The rest of the water in the bamboo moldings was further removed by drying. The weight percentage of water removed by drying was between 38.8 and 72.9 wt.%, as shown in Figure 7. Two different drying methods were used: freeze drying at −60 °C under vacuum and forced-air drying at 80 °C in the oven. During freeze drying, the water form changes from a solid state directly to a gaseous state due to the sublimation. The shape and volume of the molding material and even the ice skeleton remain unchanged. However, freeze drying required a long time, as the bamboo molding material contained a large amount of water, as shown in Figure 8. A total removal of the water in the bamboo molding materials, without previous water removal, needed 4 days, while for those pressurized materials, it needed around 3 days for freeze drying. After pressing, the bamboo roughcast had a different water content but showed similar drying characteristics since freeze drying was a gentle and slow process.

The efficiency of high-temperature air drying was much higher than that of freeze drying. All the samples reached a drying state within 14 h. Other than efficiency, the product quality was highly dependent on the drying process, which involved a complex interplay of heat and mass transfer. The heat transfers from the heating tube to the surface of the wet samples through convection and further passes into the inner part through conduction. Concurrently, water in the samples migrates from the inside part to the surface through diffusion and evaporates due to the heating medium [35]. In this process, unlike freeze drying, improper temperature and drying speed could lead to defects in samples’ structure, such as shrinkage, cracking, deformation, etc. The drying program should be carefully designed to ensure that bamboo-based porous molding materials have no defects with a proper moisture content.

### 3.5. Mechanical Performance of Bamboo-Based Porous Molding Material

**Figure 9:**
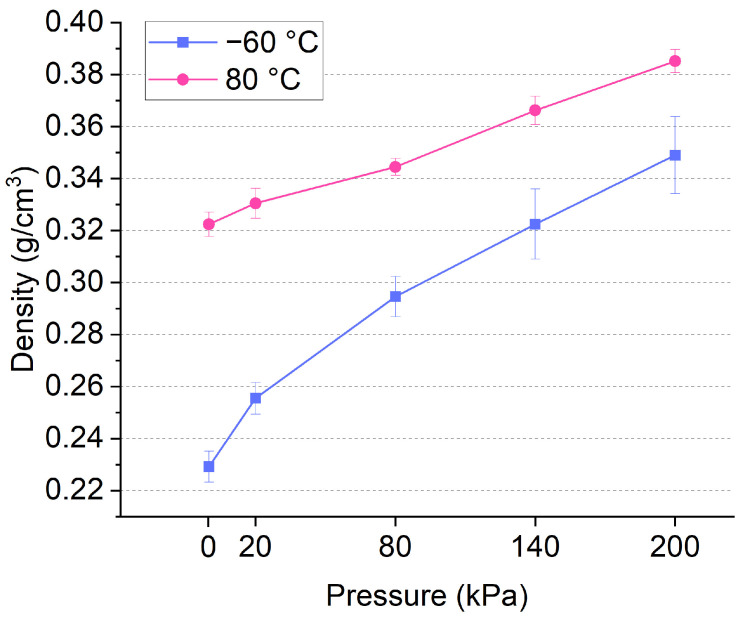
Relationship between pressure and density of bamboo-based porous molding materials under freeze drying and conventional oven drying.

The pressure applied to the molding not only removed the water but also made the molding much tighter, which resulted in an increase in the density. Figure 9 shows the relationship between the pressure and density of bamboo-based molding materials. The density of final products increased from 0.32 to 0.39 g/cm^3^ at the drying temperature of 80 °C, and from 0.23 to 0.35 g/cm^3^ at the drying temperature of −60 °C. The density of the molding material increased with the pressure in a linear trend up to a pressure of 200 kPa. Compared to bamboo, which normally has a density of 0.65–0.80 g/cm^3^, all the bamboo-based molding material has a much lower density due to its porous structure. Aiming at a lightweight material, freeze drying is preferred, as the ice skeleton is maintained during the drying process. The lightweight structure of the molding material implicates its potential applications as insulation material in the building industry.

**Figure 10:**
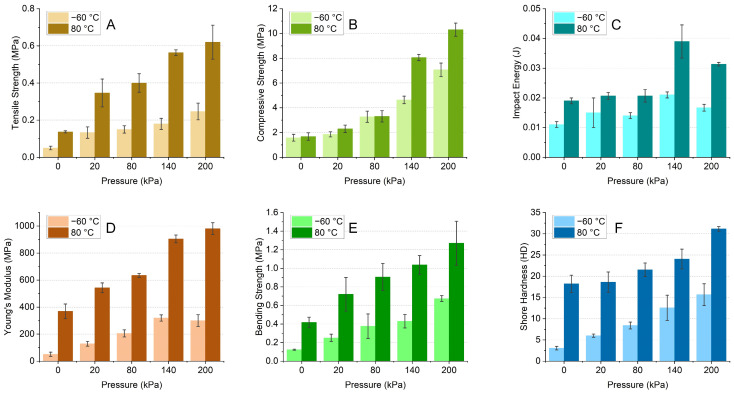
Mechanical performance of bamboo-based molding materials: (**A**) Tensile strength; (**B**) Compressive strength; (**C**) Impact energy; (**D**) Young’s modulus; (**E**) Bending strength; (**F**) Schore hardness.

Other than the density, the mechanical performance of the bamboo molding material was also significantly influenced by the water content and drying method, as shown in Figure 10. In terms of tensile strength, Young’s modulus, compressive strength, bending strength, impact energy, and shore hardness, samples obtained from a high temperature possessed superior quality compared to those obtained from freeze drying. All the performances increased with the pressure in the range of 20–200 kPa, particularly for tensile strength and compressive strength. The tensile strength increased from 0.05 to 0.25 MPa by freeze drying and from 0.14 to 0.62 MPa by oven drying as the pressure increased from 0 to 200 kPa. The compressive strength increased from 1.58 to 7.06 MPa by freeze drying and from 1.68 to 10.31 MPa by oven drying as the pressure increased from 0 to 200 kPa. The influence of pressure on the impact energy and shore hardness was smaller than that of tensile, compressive, and bending strengths.

The applications of bamboo molding materials are determined by mechanical performance to a large extent. The mechanical performance is closely related to several steps in the preparation process. During the HTG process, bamboo’s own bindings were released from lignin and hemicellulose structures. Furthermore, high temperature and pressure reactions helped to activate the binding components to generate radicals. The bamboo fibers began to disintegrate and provided the anchorage that contributed to the mechanical strength of the products. The pressure exerted on the wet roughcast reduced the gaps in the fibers together with the water removal. The drying procedure hardened the molding and gave the samples mechanical strength through water plasticization and heat plasticization. The properties of the final products could be tuned through the settings of the key preparation steps.

## 4. Conclusions

Based on the bamboo residue, we have developed a method to prepare bamboo-based porous molding materials. A hydrated thermochemical grinding process was designed to activate the bamboo’s own binding components. No synthetic adhesives were required, and the preparation process was a totally clean form of production. A suspension was formed with microscale and macroscale fibers to provide the structure and strength of the final products. The density of the dried molding samples was in the range of 0.23–0.39 g/cm^3^. The resulting suspension used as molding raw material contained around 83 wt.% water. Water was removed in two steps of pressing followed by drying. The lightweight porous samples were obtained by freeze drying at −60 °C while high-mechanical-performance samples were obtained by forced-air oven drying at 80 °C. The tensile strength and compressive strength could reach 0.62 and 10.31 MPa, respectively. The bamboo-based molding materials are fully composed of lignocellulosic components that are easy to recycle. The products molded at 80 °C are fit for high-strength applications, while those prepared by freeze drying at −60 °C can be used as thermal insulators. Future studies should include quality improvement, property assessment and modification, and industry-scale production.

## Figures and Tables

**Table 1:** Pyrolysis data of bamboo before and after HTG process.

Samp.	The First Stage		The Second Stage		The Third Stage		Resid.
	Temperature (°C)	ΔWeight (wt.%)	Temperature (°C)	ΔWeight (wt.%)	Temperature (°C)	ΔWeight (wt.%)	Weight (wt.%)
B.B. *	40–280	5.85	280–400	71.50	400–800	10.47	12.18
B.A.	40–235	4.75	235–370	58.50	370–800	14.80	21.95

*: B.B. represents bamboo before HTG process; B.A. represents bamboo after the HTG process.

## Data Availability

Data are contained within the article.
